# Pediatric Early Warning Score in interhospital ambulance care: a pilot study exploring feasibility and impact

**DOI:** 10.1186/s13049-025-01383-6

**Published:** 2025-04-18

**Authors:** Marie Luijmes, Jikke Stevens, Nicolette Diets, Rudolf Tolsma, Janke de Groot, Joris Fuijkschot

**Affiliations:** 1 Radboudumc Amalia Children’s Hospital, Nijmegen, The Netherlands; 2 Radboudumc Amalia Children’s Hospital, Canisius Wilhelmina Hospital, Nijmegen, The Netherlands; 3 Regionale Ambulance Voorziening Utrecht, Utrecht, The Netherlands; 4 Ambulance IJsselland, Zwolle, The Netherlands; 5 Knowledge Institute of the Dutch Association of Medical Specialists, Utrecht, The Netherlands

**Keywords:** Dutch PEWS, Pediatric Early Warning Score, Implementation, Interhospital ambulance care, Chain of care

## Abstract

**Background:**

Pediatric Early Warning Scores (PEWS) are commonly used for early recognition of clinical deterioration in hospitalized children and timely intervention. In 2019, a national Dutch PEWS was developed for pediatric hospital care in the Netherlands. To improve communication during interhospital transfers, using Dutch PEWS in interhospital ambulance care might be of added value in the chain of care. Therefore, this pilot study aimed to explore the feasibility and impact of the Dutch PEWS in interhospital ambulance care.

**Methods:**

Using the Plan-Do-Study-Act cycle, a four-step model for carrying out change, the Dutch PEWS system was first adopted for use in interhospital ambulance care, resulting in Dutch-Ambulance-PEWS (DA-PEWS) (Plan). This system was implemented in one ambulance region: the Regional Ambulance Service Utrecht (RAVU) (Do). Feasibility for implementing DA-PEWS nationwide and impact were evaluated. To do so, one baseline questionnaire and semi-structured interviews at the start of and at three, six, and twelve months after implementation were used (Study). Based on the results, approaches were developed to disseminate the DA-PEWS to national ambulance care (Act).

**Results:**

Main impact themes that emerged included the enhancement of situational awareness, communication in the chain of care through improvements in uniformity and handovers and improved protocol adherence. Using the system in the interhospital care setting was considered feasible, but for future upscaling of the implementation and efficacy, determinants such as variation in organizational structures, the limited frequency of pediatric interhospital transfers and differences in individual attitudes toward using one system are first steps to consider.

**Conclusion:**

This pilot study showed impact of using the DA-PEWS in interhospital ambulance care in the Netherlands, while also revealing important lessons for the implementation of the DA-PEWS nationwide due to local contextual factors in the organization of ambulance care across regions.

**Supplementary Information:**

The online version contains supplementary material available at 10.1186/s13049-025-01383-6.

What is known about this subject?

- Hospital use of PEWS may help identify children prior to their clinical deterioration and improve situational awareness among hospital staff.
- PEWS may be valuable in triage and for determining team composition for high complex interhospital transfers.

What this study adds.

- DA-PEWS seems to have added value in the interhospital transport of children by improving situational awareness of the ambulance crew as well as communication in the chain of care.
- The use of the DA-PEWS in the interhospital care setting is feasible but variations in organizational structures and the limited frequency of pediatric interhospital transfers impede implementation and need to be addressed first.

## Background

Pediatric Early Warning Scores (PEWS) have become an integral part of healthcare systems worldwide for early recognition of clinical deterioration in hospitalized children and for enabling healthcare professionals to intervene and stabilize patients in a timely manner [1]. A study conducted in 2014 identified that due to the lack of a standardized national PEWS methodology, an extensive heterogeneity of PEWS systems exists in the Netherlands, with a considerable number remaining unvalidated or self-designed [2]. In 2019, the Dutch PEWS (D-PEWS) was developed for use in pediatric hospital care in the Netherlands [3]. The D-PEWS is a uniform system that integrates a core set of vital parameters with patient responsiveness and watcher signs. Using risk stratification, outcomes are combined to a risk category to predict clinical deterioration more accurately and allow timely intervention [4, 5]. Currently, a prospective, nationwide, multicenter evaluation study is ongoing to validate the D-PEWS and assess the impact on improving patient safety in Dutch hospital care [5].

In recent years, PEWS systems have been integrated into the interhospital transport process of low-and medium complex ambulances in the Netherlands [6]. Both in the Netherlands and internationally, limited research has been conducted on the use of PEWS in ambulance care. Although some international studies have assessed the utility of PEWS-systems in prehospital practice [7, 8], few studies exist regarding the utilization of PEWS during interhospital transfers of noncritically ill patients.

Studies have shown that PEWS might be useful in triaging and making decisions on ambulance crew composition, as higher scores are associated with PICU admission and increased risks of medical interventions during transport [9–11]. Moreover, elevated transport-specific pediatric illness severity scores could predict clinical deterioration of transported patients [12]. In addition to these factors, PEWS could be a helpful tool for communication in the chain of care [10, 13].

Preliminary results from the D-PEWS study indicate that the majority of pediatric physicians and nurses see benefits of uniform use of the D-PEWS in the chain of care, including monitoring the clinical course, handovers, situational awareness and quality of care (Additional file 1, Tables 1, 2 and 3) [5].

This pilot study aimed to investigate the feasibility and the impact of using the D-PEWS in interhospital ambulance care of noncritically ill patients in the Netherlands in order to explore the potential of implementing these systems in the ambulance sector. Knowledge gained by this research can be used in future research on the use of PEWS systems in the context of ambulance care.

## Methods

### Aim

Explore feasibility and the impact of using the D-PEWS in interhospital ambulance care of non-critically ill patients.

### Study design

**Fig. 1:**
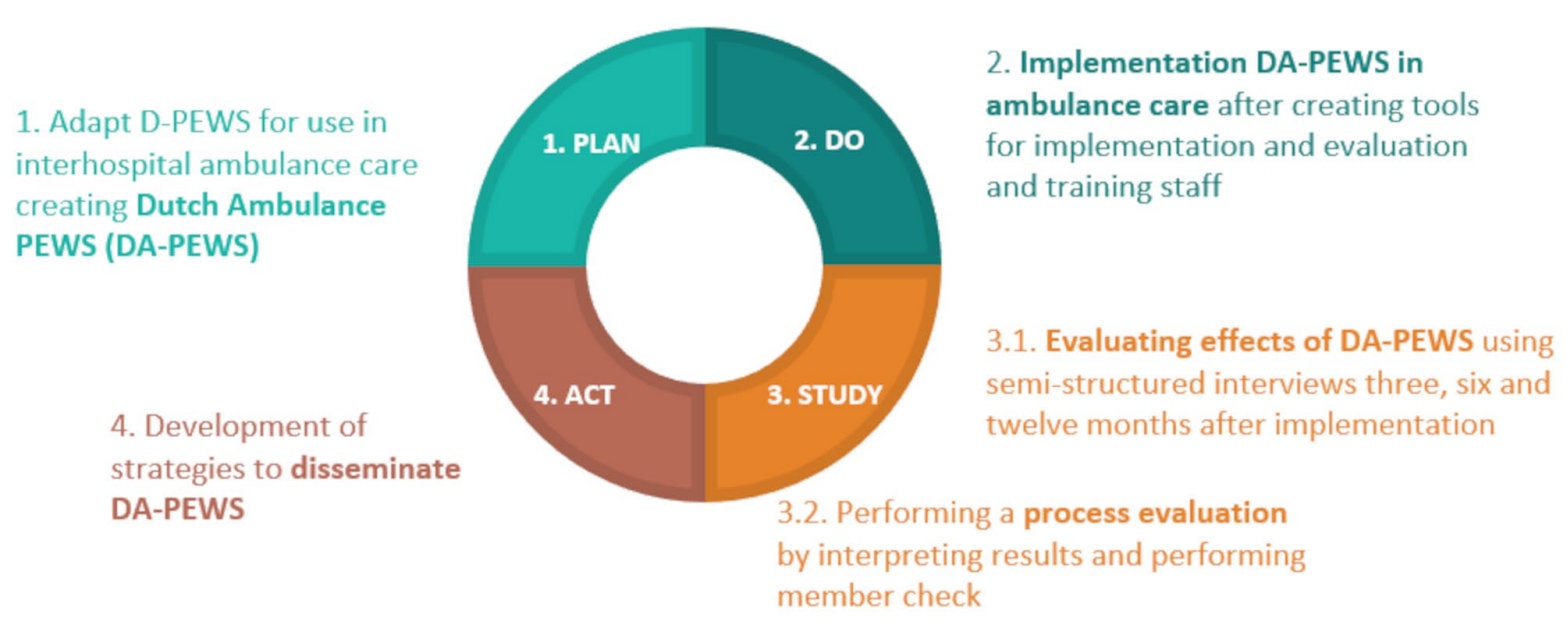
Plan-Do-Study-Act cycle

The methods consisted of a pilot study in which qualitative methods were used to evaluate the implementation in one strategically chosen regional ambulance service. For integration of the D-PEWS in this ambulance organization, the four-step model for carrying out change is used: the Plan-Do-Study-Act cycle (PDSA) [14] (Fig. 1). The content of the PDSA cycle is explained in detail in later sections.

#### Setting

The ambulance care in the Netherlands is accountable for interhospital transfers and is regionally organized into 25 Regional Ambulance Services (RAVs). The Regional Ambulance Service Utrecht (RAVU) is accountable for the Province of Utrecht. The RAVU is organized into three levels of care (Table 1).

**Table 1:** Levels of care at the Regional Ambulance Service utrecht

Level of care	Transport	Education
High complex (HC) ambulances	Specialized in Advanced Life Support. Treatment and transport of complex medical cases including ICU transfers	Bachelor of Science in Nursing (with additional education in the ambulance field (theoretical and practical training) to become an ambulance nurse)
Medium complex (MC) ambulances	Cardio-respiratory stable patients with care issues that are non-life threatening	Secondary vocational education (MBO)
Low complex (LC) ambulances	Low complex issues (predictable, non-life-threatening situations)	Secondary vocational education (MBO)

The ambulance crew typically consists of one specialized nurse and one driver (with more limited training and qualifications).

For this pilot study we chose the RAVU because this region houses two highly specialized children’s hospitals, the Princess Maxima National Center for Pediatric Oncology (PMC), and the University Wilhelmina Children’s Hospital (WKZ). Both PMC and WKZ implemented the D-PEWS as part of the national study [5]. Implementing the D-PEWS in interhospital ambulance care in this region enables the possibility of studying the complete chain of care, in which transports are not only directed to specialized centers but also to general hospitals and large teaching hospitals. In June 2020, as a pilot, the PEWS-system of the PMC was partly incorporated at the RAVU prior to the D-PEWS.

### PDSA-Cycle

#### Plan

##### The Dutch-ambulance-PEWS (DA-PEWS)

The original hospital D-PEWS was adjusted for application in ambulance care in collaboration with the RAVU under guidance of the local project lead (ND) creating the Dutch-Ambulance-PEWS (DA-PEWS) (Additional file 2, Table 1) [4]. The layout, risk-categories, scoring of vital parameters and watcher signs were identical to the D-PEWS. Adjustments were made in the actions following the PEWS and cutoff values for transport and escalation of care to fit current ambulance protocols [6].

##### Inclusion and exclusion criteria

The inclusion criteria for transport with the DA-PEWS include scheduled interhospital transport of cardiorespiratory stable patients executed by the MC-ambulances:

- Aged 3–18 years from the PMC
- Aged 6–18 years from and to other hospitals

The exclusion criterium was the scheduled ambulance transport of terminal palliative patients executed by the MC-ambulances. Younger age groups are excluded because these transports are conducted by HC-ambulances, as stipulated by the national ambulance protocol. Except for the PMC due to the previous pilot in which the age limit was lowered.

##### Outcome measures

Predefined outcome measurements originated predominantly from the multicenter D-PEWS study and were based on Endsley’s Situational Awareness (SA) framework [5, 15]. This model explains how individuals and groups perceive, comprehend and anticipate information in dynamic task environment. It is described according to three levels: (1) perception of elements and information in the environment, (2) interpretation of that information and understanding a situation and (3) projection and anticipation of future status and events.

Outcome measures:

1. Impact - effects of DA-PEWS on quality of care by improving:

1. Situational awareness; general effects of SA and effects of SA upon arrival at the referring institute and during transport;
2. Chain of care; the effects of DA-PEWS on quality of care within the chain (from referring hospital to ambulance service to the receiving hospital), including the effects on communication and handovers;
3. Quality of care; general effects regarding the quality of care, including protocol adherence.

2. Feasibility - barriers and facilitators at:

1. Local level (RAVU);
2. National level.

##### Baseline measurement

A baseline measurement (T = 0) was conducted among staff using a questionnaire focusing on experiences and perceptions of the staff with their current PEWS-system. Questions were categorized according to the outcome measures and were presented as statements using a 7-point Likert Scale, for example ‘The PEWS contributes to monitoring the course of vital signs during interhospital transport’ and ‘The PEWS contributes to effective communication within the RAVU’. Participants for the semi-structured interviews were recruited by sending a registration list via e-mail to all staff members of the MC-ambulances. The goal was to conduct six interviews. All participants signed informed consent prior to participation. Interviews focused on exploring personal experiences with and benefits plus disadvantages of the PEWS-system. The interviews, which lasted approximately 30 min, were conducted by ML and were audio recorded for subsequent analysis.

#### Do

##### Preparations for implementation

Tools for implementation and evaluation were developed in codesign with the RAVU (ND). It consisted of (1) a description of the DA-PEWS system, including pocket cards and a free-accessible instruction video; (2) evaluation tools (consisting of a questionnaire and interview guides); and (3) a transportation evaluation form to document experiences of employees with the system, which they completed manually on paper after each transfer. The questions from the questionnaire and semi-structured interview-guides were based on the outcome measures and literature [16, 17]. The interview-guides contained a list of questions addressing different topics to impose structure on data collection.

At T = 0 the DA-PEWS was implemented at the RAVU after training the staff using a presentation explaining the instrument, the research and including a practical session.

#### Study

##### Evaluation of working with the DA-PEWS in MC-ambulances

Staff of MC-ambulances were interviewed at three, six and twelve months (T = 3, T = 6, T = 12) after implementation. During the study period, the transportation evaluation form was used to document the frequency of DA-PEWS usage as an indicator of its implementation. At T = 3, the interviews focused on experiences relating to the implementation process. At T = 6 and T = 12, we focused on the perceived impact of the DA-PEWS on pediatric ambulance care. Questions that were used included, for example ‘How did the implementation of the DA-PEWS proceed?’, ‘In your experience, what is the role of the DA-PEWS in identifying critically ill children?’ and ‘What do you believe the effect of risk stratification on PEWS?’. Participants for the interviews were recruited by sending emails to the staff and by purposive sampling based on previous interview experience.

##### Process evaluation

The subsequent process evaluation to help interpret results and perform a member check was augmented by meetings with stake holders of the RAVU and national ambulance care services.

#### Act

Based on the findings, strategies were developed to disseminate the DA-PEWS to other ambulance services and to scale up the project.

### Data management

The interviews were transcribed verbatim. Data saturation was measured and defined as the point at which no new themes or perspectives emerged. All interviews were analyzed with Atlas.ti v8 software and coded using Qualitative Content Analysis [18, 19] by researcher ML. The text was divided into meaning units (quotations) containing relevant information. The framework of SA according to Endsley was used for thematic coding [15, 16]. The levels of SA were divided into effects upon arrival at the referring hospital, during transport, and effects applicable for both arrival at the hospital and during transport, which mainly included effects on individual level. Complex quotations were evaluated with D-PEWS researcher JS to increase the reliability of the analysis. The results from the questionnaire were processed using Excel.

## Results

The characteristics of the participants are described in Table 2.

**Table 2:** Characteristics and number of participants

	Number of participants in relation to all staff	Percentage of participants	Years of work experience		
			< 1 year	1–5 years	> 5 years
**Questionnaire**					
T = 0	27 (15 nurses, 12 drivers MC) / 30	90%	12	6	9
**Interviews**					
T = 0	4 nurses MC / 30	13%	0	3	1
T = 3	4 nurses MC / 20	20%	0	3	1
T = 6	3 nurses MC / 20	15%	1	1	1
T = 12	3 nurses (1 nurse LC, 2 nurses MC) / 55 (35 LC staff members, 20 MC staff members)	5%	0	2	1

Number of participants refers to the number of participants in relation to the total number of staff of the MC and LC-ambulances. % Refers to the percentage of participants in relation to number of personnel

### Plan & Do: Baseline measurement and currently used PEWS

#### Questionnaire

Comprehensive information regarding the statements used and results from the questionnaire are detailed in Additional file 3, Tables 1, 2 and 3. The main result from the questionnaire is that participants– based upon previous experience– generally perceive PEWS as a valuable tool (Table 3). It is considered beneficial for situational awareness, the chain of care, protocol adherence and safety. The suggestions for improvement consisted of more uniformity and improved clarity regarding agreements on protocol.

**Table 3:** Impact Pediatric Early Warning Score - questionnaire T = 0

Theme	Sublevel	Result
SA	Upon arrival and during transport	Insight course of vital signs Improvement situational awareness
	Concerns healthcare provider/parents	There is room for concerns of the healthcare provider within PEWS. Fewer participants agreed with the statement that there is room for parental concerns within PEWS
Chain of care	Communication and handovers	Contribution of efficient handovers and effective communication A uniform system would optimize communication and handover
General	Operational procedure	Clear guidelines regarding PEWS Feasible to adhere to agreements
	Safety	PEWS contributes to safe transfer of patients
Rating of current PEWS		8.2/10
Suggestions for improvement for DA-PEWS (open question)		Themes (percentage of answers): More uniformity (58%) Improved clarity regarding agreements and protocol (33.3%)

#### Interviews

Three key themes and multiple subthemes were identified across the interviews (Table 4). An overview of the themes and subthemes, including quotes, is provided in Additional file 4, Tables 1, 2 and 3. We were able to recruit four participants for the interviews. This number of interviews was sufficient to reach data saturation. The effects of PEWS were mostly related to SA level 1 (perception of relevant information). Codes associated with SA level 2 (comprehension and interpretation of the relevant information) were less frequent. No codes relating to SA level 3 (projection of future status and events) arose from the interviews [15].

**Table 4:** Impact of Pediatric Early Warning Score– before implementation (T = 0)

Theme	Sublevel	Effect
SA level 1	Individual	Increased awareness of vital signs Aged-based reference range for vital signs Importance of gut feeling nurse/parents (position and usage disputed among interviewees)
	Upon arrival	During handover a PEWS-trend can provide insight on the course of a patient’s condition during the hospitalization PEWS enforces decision making regarding transport safety and proper team composition
	During transport	PEWS helps creating a trend on patients’ condition during transport Provision of clear agreements on escalation of care
SA level 2	Individual	PEWS can aid in objectifying clinical judgment by assigning numeric value to clinical situations PEWS helps in the interpretation of vital signs
Chain of care	Uniformity	Lasting desire for uniformity between ambulance service and hospitals Speaking the same language with nurses from PMC (using the same PEWS-system)
	Communication and handovers	PEWS not always automatically mentioned during handovers, but when used it provides added value to efficient and clear handovers
General	Protocol adherence	Guidance on and adherence to protocol Helps in the decision and execution of escalation of care based on protocol

### Study: Evaluation of Dutch-Ambulance-PEWS at the RAVU

During the study period, an unexpected limited number of pediatric transfers were executed by the MC-ambulances, limiting exposure and practical experience with the DA-PEWS.

The research group attempted to increase exposure by expanding the study period and including LC-ambulances– after completing the educational program in April 2022. The total study period spanned from December 2020 to December 2022. MC ambulances transported approximately 240 children during this period, however due to central registration issues at the location, an insufficient number of transportation evaluation forms were recorded. Therefore, the exact number of transports using DA-PEWS is unknown. Moreover, we were unable to use this data regarding employees’ experiences after each transfer.

#### Interviews

An overview of the themes and subthemes, including quotes is provided in Additional file 5, Tables 1, 2, 3 and 4. During the interviews, we did not achieve complete data saturation. We attempted to recruit more participants, however, despite purposeful sampling, responses were limited.

##### Impact

The impact of the DA-PEWS on SA was mainly related to improvement of SA level 1 and to a lesser degree of level 2. In comparison with the previously used PEWS system, there are similar benefits but also additional effects of the DA-PEWS, highlighted in Table 5 using bold text. The participants were positive about specific elements of the DA-PEWS, including the worried sign, risk stratification and the increased uniformity within the chain of care.

**Table 5:** Impact the Dutch Ambulance Pediatric Early Warning Score– after implementation

Theme	Sublevel	Effect
SA level 1	Individual	**Worried sign as important contribution for obtaining overview on patients’ condition** Increased awareness on vital signs Aged-based reference range for vital signs **Pocket-cards as helpful tool**
	Upon arrival	**Risk stratification to evaluate patients’ status and making a risk assessment prior to transportation** PEWS enforces decision making regarding transport safety and proper team composition
	During transport	Helps creating a trend on patients’ condition during transport **Risk stratification to evaluate patients’ status and evaluate need to escalate care according to protocol** Provision of clear agreements on escalation of care
SA level 2	Individual	PEWS can aid in objectifying clinical judgment by assigning numeric value to clinical situations PEWS helps in the interpretation of vital signs
	**Upon arrival**	**Guidelines on when to consult with their medical manager for interpretation of patients’ condition**
	**During transport**	**Assistance in interpreting a deteriorating trend during transport**
Chain of care	Uniformity	**Increased uniformity by speaking the same language** Lasting need for uniformity between ambulance service and hospitals as not all hospitals use D-PEWS
	Communication and handovers	**Uniform communication using D-PEWS** **Improved handovers**,** particularly to the team of the receiving hospital as it helps to describe clinical course during transport**
General	Protocol adherence	Guidance on and adherence to protocol Cut-of values for transportation and escalation of care

New effects of the DA-PEWS are highlighted using bold text

##### Feasibility

Multiple barriers to and facilitators of the implementation process were identified by the participants (Additional file 5, Table 4). The most frequently mentioned barriers and facilitators are described in Table 6.

**Table 6:** Feasibility - after implementation

Barriers	Perceived limited number of pediatric transfers executed by the MC and LC-ambulancesLack of background knowledge on the protocol of the DA- PEWS
Facilitators	Educational training Pocket cards
Dissemination	Suitable for other ambulance services and for transport process of MC- and LC-ambulances, provided getting the same introduction and implementation as at the RAVU

### Act: Dissemination 

While this was a pilot study looking at feasibility, we did encounter exposure issues that could hinder future research. Implementing and evaluating DA-PEWS in other RAVs could address these low exposure issues. There have been meetings with the local project lead from the RAVU (ND) and three stakeholders, including a project manager from AZN (Ambulance Care Netherlands, national organization for ambulance care) and two experts in the field from different RAVs, including a Manager of Ambulance Care and a Nurse Practitioner Emergency Medicine (RT). Despite these meetings, we were not able to recruit more RAVs for implementing DA-PEWS, mainly because of differences in local organizational structure. Additionally, stakeholders had varied ideas regarding the use and impact of a national PEWS for interhospital care for LC- and MC-ambulances. Therefore, before scaling up research, it is important to carefully study and address the national determinants that influence implementation.

## Discussion

This pilot study examined the impact and feasibility of DA-PEWS in interhospital transport. Despite relatively limited practical experience with DA-PEWS, the ambulance staff showed a positive attitude toward the use of the instrument. Moreover, questionnaires of hospital staff show that there is a predominantly positive view on the uniform use of D-PEWS in the chain of care.

### Impact

Consistent with prior research on PEWS in hospital settings, DA-PEWS seems to enhance SA [16]. Previous research has indicated that PEWS can standardize the assessment of clinical patients prior to the transport or during transport when recalculated [10]. We found that enhancing SA improved understanding of the situation and protocol adherence through accurate clinical assessment upon arrival at the hospital and during transport. The worried sign contributes to assessing a clinical situation, and risk stratification guides decisions on transportation and escalation of care. In line with previous studies [10, 13], using the DA-PEWS may optimize communication through a uniform system and thereby improve handovers in the chain of care.

### Feasibility

Using the system in interhospital ambulance care at one ambulance service was considered feasible, however, the main barrier for implementation is the limited number of pediatric transports perceived by staff, resulting in limited exposure and potential secondary barriers. Further dissemination was hindered by variations in organizational structures among ambulance services, the limited number of transports and differing views on the potential added value.

### Methodological considerations/ limitations

Unfortunately, due to registration issues, we were unable to identify the exact number of pediatric transfers using DA-PEWS and therefore determine the level of implementation. To gain better insight into the use of the system, it would be more effective to use automatic registrations. Although this was not possible during this pilot, future efforts could focus on integrating the DA-PEWS into the electronic patient record. During the interviews, we did find that participants used the DA-PEWS during every pediatric transfer fitting the inclusion criteria. However, they perceived a limited number of pediatric transfers. The impact of DA-PEWS therefore remains partly based on expected benefits from professionals. Furthermore, we were unable to expand the research and demonstrate its generalizability to other ambulance services.

Nevertheless, it could be plausible that the results from the pilot, such as the effects on SA, are generalizable to similar ambulance contexts and other ambulance transports as these are general concepts, both within the Netherlands and internationally. Moreover, the knowledge generated from the PDSA cycle can be valuable for other implementation of PEWS-systems in ambulance care. Future research potential may lie in initiating collaborations at the national level of ambulance care organizations. Understanding the factors contributing to differing viewpoints on potential added value among professionals is essential.

An additional limitation of the study is that during the interviews of the evaluation, while we were able to attain a comprehensive understanding of the perspective of the participants, we did not achieve complete data saturation. Selection bias might have occurred in the interviews as enthusiastic, receptive personnel volunteered. It is possible that we might have missed other perspectives or contradictory opinions. Nevertheless, the results from T = 0 from the questionnaire with a high response rate were largely in line with the results from the interviews.

## Conclusion

In conclusion, this pilot study suggests that implementation of the DA-PEWS might impact interhospital care beneficially, but this requires overcoming some significant barriers hindering national implementation first. Further research is needed to identify how these barriers can be overcome.

## Electronic supplementary material

Below is the link to the electronic supplementary material.

Supplementary Material 1

Supplementary Material 2

Supplementary Material 3

Supplementary Material 4

Supplementary Material 5

## Data Availability

The datasets used and/or analyzed during the current study are available from the corresponding author upon reasonable request.

## Abbreviations

D-PEWS: Dutch PEWS
DA-PEWS: Dutch Ambulance PEWS
HC: High complex
LC: Low complex
MC: Medium complex
PDSA-cycle: Plan-Do-Study-Act cycle
PEWS: Pediatric Early Warning Scores
PICU: Pediatric Intensive Care Unit
PMC: Princess Maxima Center
RAV: Regional Ambulance Services
RAVU: Regional Ambulance Service Utrecht
SA: Situational Awareness
WKZ: Wilhelmina Children’s Hospital
