# Supplementary material for: Pediatric Early Warning Score in interhospital ambulance care: a pilot study exploring feasibility and impact
Source: Scand J Trauma Resusc Emerg Med. 2025 Apr 18;33:65. doi: 10.1186/s13049-025-01383-6 (PMC12007274; doi:10.1186/s13049-025-01383-6)
Supplement: Supplementary file 1 — Supplementary Material 1 [file 13049_2025_1383_MOESM1_ESM.docx]

# Additional file 1. Supporting information on results from questionnaire regarding Dutch PEWS in hospitals

**Table 1.** Participants and years of work experience questionnaire

| **Answer choices** | **Occupation** | **Number of participants** | **Years of work experience** | | | |
| --- | --- | --- | --- | --- | --- | --- |
|  | | | 0-5 years | 5-10 years | 10-20 years | >20 years |
| T=6 | Doctor | 62 | 34% | 23% | 31% | 13% |
|  | Nurse | 184 | 27% | 19% | 17% | 37% |
| T=12 | Doctor | 83 | 27% | 23% | 28% | 23% |
|  | Nurse | 194 | 26% | 16% | 20% | 39% |

**Table 2.** Results from questionnaire T =6

Doctors

| **Statements** | **Response** | **Strongly agree** | **Agree** | **Partly agree** | **Neutral** | **Partly disagree** | **Disagree** | **Strongly disagree** |
| --- | --- | --- | --- | --- | --- | --- | --- | --- |
| Uniform use of the Dutch PEWS enhances the monitoring of the clinical course of a child, thereby improving the quality of care across the chain of care | 50 | 8% | 28% | 28% | 28% | 4% | 2% | 2% |
| Uniform use of the Dutch PEWS contributes to the quality of handovers during transfers across the chain of care | 50 | 8% | 24% | 34% | 26% | 4% | 4% | 0% |
| Uniform use of Dutch PEWS enhances the situational awareness of the team in the receiving hospital in the chain of care | 49 | 6% | 24% | 39% | 27% | 0% | 4% | 0% |
| The implementation of the Dutch PEWS at ambulance services and its application during interhospital transfers is of added value for ensuring the quality of care during patient transfers. | 50 | 6% | 28% | 30% | 26% | 8% | 2% | 0% |

Nurses

| **Statements** | **Response** | **Strongly agree** | **Agree** | **Partly agree** | **Neutral** | **Partly disagree** | **Disagree** | **Strongly disagree** |
| --- | --- | --- | --- | --- | --- | --- | --- | --- |
| Uniform use of the Dutch PEWS enhances the monitoring of the clinical course of a child, thereby improving the quality of care across the chain of care | 147 | 7% | 43% | 20% | 18% | 7% | 5% | 1% |
| Uniform use of the Dutch PEWS contributes to the quality of handovers during transfers across the chain of care | 147 | 8% | 45% | 17% | 18% | 9% | 3% | 0% |
| Uniform use of Dutch PEWS enhances the situational awareness of the team in the receiving hospital in the chain of care | 147 | 10% | 43% | 19% | 20% | 7% | 1% | 0% |
| The implementation of the Dutch PEWS at ambulance services and its application during interhospital transfers is of added value for ensuring the quality of care during patient transfers. | 147 | 9% | 40% | 22% | 21% | 6% | 2% | 0% |

**Table 3.** Results from questionnaire – T=12

Doctors

| **Statements** | **Response** | **Strongly agree** | **Agree** | **Partly agree** | **Neutral** | **Partly disagree** | **Disagree** | **Strongly disagree** |
| --- | --- | --- | --- | --- | --- | --- | --- | --- |
| Uniform use of the Dutch PEWS enhances the monitoring of the clinical course of a child, thereby improving the quality of care across the chain of care | 68 | 6% | 29% | 21% | 37% | 3% | 2% | 3% |
| Uniform use of the Dutch PEWS contributes to the quality of handovers during transfers across the chain of care | 68 | 3% | 37% | 18% | 28% | 3% | 9% | 3% |
| Uniform use of Dutch PEWS enhances the situational awareness of the team in the receiving hospital in the chain of care | 67 | 21% | 33% | 25% | 31% | 2% | 3% | 3% |
| The implementation of the Dutch PEWS at ambulance services and its application during interhospital transfers is of added value for ensuring the quality of care during patient transfers. | 68 | 3% | 28% | 27% | 29% | 6% | 4% | 23% |

Nurses

| **Statements** | **Response** | **Strongly agree** | **Agree** | **Partly agree** | **Neutral** | **Partly disagree** | **Disagree** | **Strongly disagree** |
| --- | --- | --- | --- | --- | --- | --- | --- | --- |
| Uniform use of the Dutch PEWS enhances the monitoring of the clinical course of a child, thereby improving the quality of care across the chain of care | 150 | 9% | 41% | 18% | 21% | 7% | 3% | 1% |
| Uniform use of the Dutch PEWS contributes to the quality of handovers during transfers across the chain of care | 148 | 9% | 45% | 16% | 18% | 6% | 5% | 1% |
| Uniform use of Dutch PEWS enhances the situational awareness of the team in the receiving hospital in the chain of care | 149 | 8% | 42% | 16% | 24% | 75% | 4% | 1% |
| The implementation of the Dutch PEWS at ambulance services and its application during interhospital transfers is of added value for ensuring the quality of care during patient transfers. | 150 | 9% | 40% | 15% | 25% | 5% | 5% | 1% |
