# Supplementary material for: Pediatric Early Warning Score in interhospital ambulance care: a pilot study exploring feasibility and impact
Source: Scand J Trauma Resusc Emerg Med. 2025 Apr 18;33:65. doi: 10.1186/s13049-025-01383-6 (PMC12007274; doi:10.1186/s13049-025-01383-6)
Supplement: Supplementary file 2 — Supplementary Material 2 [file 13049_2025_1383_MOESM2_ESM.docx]

# Additional file 2. Dutch PEWS and Dutch Ambulance PEWS

**Figure 1.** Infographic Dutch PEWS


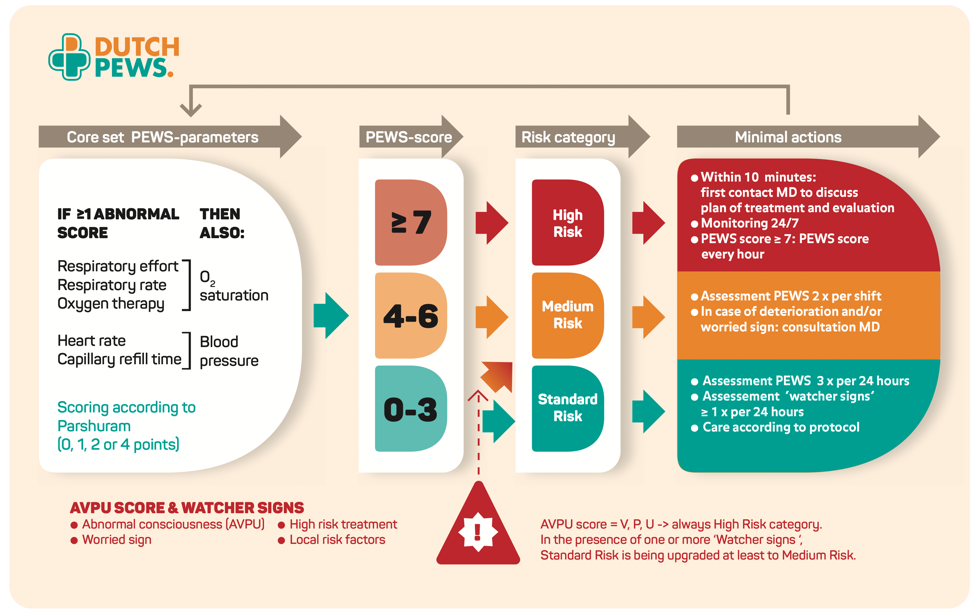


**Table 1.** Dutch ambulance PEWS

| Risk category | PEWS Score | Applicable ‘watcher signs’ | Actions |
| --- | --- | --- | --- |
| High Risk | ≥ 7 points | Worried sign + deteriorating PEWS  AVPU = V, P or U | Emergency assistance from HC-ambulance  Contact attending physician at referring hospital |
| Medium Risk | 4 – 6 points | Worried sign  AVPU = A | Assessment PEWS, worried sign and AVPU at least every 10 minutes during transport  Contact attending physician at referring hospital  Contact the Medical Manager from the RAVU |
| Standard Risk | 0 – 3 points | No Worried Sign  AVPU = A | Care according to protocol  Assessment PEWS, AVPU, worried sign at least every 30 minutes during transport |

Based upon PEWS, AVPU and worried sign, all patients are categorized into risk categories prior to transportation. Only standard risk patients are to be transported by the Medium Care-ambulances of the Regional Ambulance Service Utrecht (as standard operating procedure). During transport, PEWS, AVPU and worried sign are assessed at given time points. If a risk category changes during transport, personnel should act according to ‘Actions’.

Worried sign: parents or health care professionals have considerable concerns about the clinical course of a patient.

AVPU: a straightforward scale that is used to rapidly grade patients’ level of consciousness, responsiveness or mental state.

A = Alert, V = Verbal, P = Pain, U = Unresponsive.

Deteriorating PEWS: the patient shifts towards a higher risk category, based upon the PEWS (viral parameters) alone

HC-ambulance: High care ambulances, specialized in Advanced Life Support. It is equipped with more advanced life support facilities and high educated personnel than a medium complex ambulance.
