# Supplementary material for: Pediatric Early Warning Score in interhospital ambulance care: a pilot study exploring feasibility and impact
Source: Scand J Trauma Resusc Emerg Med. 2025 Apr 18;33:65. doi: 10.1186/s13049-025-01383-6 (PMC12007274; doi:10.1186/s13049-025-01383-6)
Supplement: Supplementary file 3 — Supplementary Material 3 [file 13049_2025_1383_MOESM3_ESM.docx]

# Additional file 3. Supporting information on results from questionnaire baseline measurement

**Table 1.** Years of work experience participants questionnaire

| **Answer choices** | **Percentages** |
| --- | --- |
| 0-1 years | 44.44% |
| 2-3 years | 22.22% |
| 4-5 years | 0% |
| >5 years | 33.33% |

**Table 2.** Results from questionnaire T=0 – statements regarding PEWS

| **Statements** | **Strongly agree** | **Agree** | **Partly agree** | **Neutral** | **Partly disagree** | **Disagree** | **Strongly disagree** |
| --- | --- | --- | --- | --- | --- | --- | --- |
| 1. The agreements applicable to each PEWS-score are clear to me. | 22.22% | 55.56% | 18.5% | 3.7% | 0% | 0% | 0% |
| 2. The agreements regarding patients with a PEWS above the alarm threshold are clear to me. | 33.33% | 51.85% | 7.41% | 7.41% | 0% | 0% | 0% |
| 3. It is feasible for me to adhere to the agreements concerning a PEWS score above the alarm threshold. | 29.63% | 48.15% | 4.7% | 18.52% | 0% | 0% | 0% |
| 4. The agreements concerning patients deteriorating during transport are clear to me. | 29.63% | 55.56% | 11.11% | 3.7% | 0% | 0% | 0% |
| 5. The PEWS contributes to the timely identification of a deteriorating patient. | 29.63% | 55.56% | 11.11% | 0% | 3.7% | 0% | 0% |
| 6. Over the past six months, I have witnessed a patient unexpectedly deteriorate during a transfer and PEWS promptly alerted in this regard. | 20% | 20% | 20% | 20% | 0% | 20% | 0% |
| 7. The PEWS contributes to monitoring the course of vital signs during interhospital transport. | 25.93% | 55.56% | 18.52% | 0% | 0% | 0% | 0% |
| 8. The PEWS adds value to creating a situational overview of patients during interhospital transport. | 11.11% | 81.48% | 7.41% | 0% | 0% | 0% | 0% |
| 9. The PEWS contributes to an efficient transfer to and from hospitals. | 29.63% | 48.15% | 14.81% | 7.41% | 0% | 0% | 0% |
| 10. The PEWS contributes to effective communication within the RAVU. | 29.63% | 48.15% | 11.11% | 11.11% | 0% | 0% | 0% |
| 11. The PEWS supports effective communication between the RAVU (Regional Ambulance Service Utrecht) and other parties, such as hospitals. | 29.63% | 51.85% | 7.41% | 11.11% | 0% | 0% | 0% |
| 14. Over the past six months, I have witnessed a decision made at the patient's bedside not to transport the patient due to safety and/or suitability concerns and PEWS aided in making the decision | 33.33% | 41.67% | 8.33% | 8.33% | 0% | 0% | 8.33%% |
| 16. A standardized PEWS system would optimize communication and handover regarding patient transfers between healthcare facilities. | 66.67% | 25.93% | 3.7% | 3.7% | 0% | 0% |  |
| 17. Within PEWS, there is a room for my concerns regarding a patient's deteriorating clinical condition. | 14.81% | 59.26% | 11.11% | 14.81% | 0% | 0% | 0% |
| 18. Within PEWS, there is room for parents' concerns about a patient's deteriorating clinical condition. | 14.81% | 33.33% | 22.22% | 22.22% | 3.7% | 3.7% | 0% |
| 19. The PEWS contributes to a safe transfer of patients | 37.04%% | 55.56% | 7.41% | 0% | 0% | 0% | 0% |

**Table 3.** Results from questionnaire – open questions regarding PEWS

| **Question 20: What are your desires a PEWS system?** | | |
| --- | --- | --- |
| **Theme** | **N** | **Responses** |
| Uniformity | 6 | - Uniformity among chain partners  - Uniformity. Currently, in one center, various PEWS scores are applicable to different age categories  - That PEWS is used everywhere in all institutions  - Nationally standardized PEWS scores and values. It varies by region/institution  - A uniform system  - Indeed, all parties follow one guideline |
| Clarity on agreements and protocol | 4 | - Clear guidelines that are not open to discussion. More experience with children, it occurs very rarely  - Clarity  - Something more specific regarding age. We have a PEWS card from the Princess Maxima Center, where an age distribution has been made. It may be a bit extensive, but within the pediatric ages, vital values vary  **-** A clear and unambiguous policy defining the limits of when we can and cannot take action |
| More experience | 2 | - Experience  - Clear guidelines that are not open to discussion. More experience with children, it occurs very rarely |
| Other | 1 | - Room for concerns/gut feeling |
| **Question 21: Do you have any suggestions for improvement or tips?** | | |
| **Theme** | **N** | **Responses** |
| Uniformity | 2 | - Explanation to, for example, the A car (High Complex ambulance) regarding some misunderstandings related to transportation  - Uniformity within care |
| Other | 1 | I do not observe attitude and behavior reflected on the pocket card. This particularly conveys information about the well-being of the child, especially in the case of younger children. Additionally, I miss the skin color on the card. Perhaps not essential for the transportation we undertake, but nationally indispensable |

|  |
| --- |
