# Supplementary material for: Pediatric Early Warning Score in interhospital ambulance care: a pilot study exploring feasibility and impact
Source: Scand J Trauma Resusc Emerg Med. 2025 Apr 18;33:65. doi: 10.1186/s13049-025-01383-6 (PMC12007274; doi:10.1186/s13049-025-01383-6)
Supplement: Supplementary file 4 — Supplementary Material 4 [file 13049_2025_1383_MOESM4_ESM.docx]

# Additional file 4. Supporting information on results from interviews baseline measurement

**Table 1.** Results from semi-structured interviews relating to effects on Situational Awareness (SA) – before implementation (T=0) of Dutch Ambulance PEWS

| **Theme** | **Sublevel** | **N** | **Quote** |
| --- | --- | --- | --- |
| **Situational awareness 1** | | | |
| Individual | Alertness to vital signs | 18 | *"Yes, that you very consciously observe the child, the vital functions, what is a child's respiration, whether there's increased respiratory effort, whether there... You just look much more attentively, before transporting the child you observe the child."* |
|  | Aged-based reference to vital signs | 15 | *“Having a reference value to assess, like if someone has a much higher respiratory rate or a much higher pulse than what is typical for that age group, I'm thinking, well, this, for me, is an alarm signal.”* |
|  | Parental sense of unease  Not included in PEWS  Included in PEWS | 14  8  5 | *“Besides that, you also have the mother who you’re keeping an eye on and like, looking for her input. It might not be written on the card, but it does complete the picture.”* |
|  | Healthcare providers’ sense of unease  Not included in PEWS  Included in PEWS | 10  7  3 | *“And especially if you already have quite a bit of experience in healthcare. Yes, a sense of unease can actually trigger you, prompt you to maybe perform those checks one more time, you know. I think it's something that certainly shouldn't be trivial; it should carry weight.”* |
|  | Contribution of pocket reference cards | 4 | *“Yes, and then you perform those assessments and you link those to the values and then I look at my card because often, like I said: I don't frequently deal with transporting such children, so I need my card. And then you add up those scores, and if it's done correctly because the hospital knows, Princess Maxima in this case as well, they shouldn't score too high, according to the LPZ scores (national protocol). And then, well, if it falls within the margin, it's not a problem."* |
| Upon arrival | Insight on the course of the condition of the patient. | 5 | *“You can also see that in the PEWS. An improvement is evident there, as the department has already conducted the PEWS. I always find it reassuring as we can then observe: hey, there's an upward trend. Or conversely, there's a downward trend. But, there's an ascending trajectory, well then, the treatment is effective and we can safely transport the child. The anticipation is that for the next hour, for instance, no unexpected developments are anticipated."* |
|  | Health care provider experience | 5 | *“That's what I consider a part of transporting the child... If you feel secure about it yourself, you also radiate that to the parent. You shouldn't leave with uncertainty yourself. So, for me, that's a very crucial point, that I reassure myself that everything is well."* |
|  | Agreements on transport | 5 | *“Well, it's about becoming aware of the values you measure. Being alert to whether it's within the expected range for the child or not, whether it's deviating. If it deviates from the agreements set through the PEWS, then you have a guideline there. Then you can also decide: 'I will proceed with this' or 'I will not proceed; I'll first consult with my medical manager'."* |
|  | Insufficient information prior to arrival | 5 | *" The dispatch center inputs certain information, and we receive a condensed version of it in our notepad. So, that's the only thing you know. And if I still have uncertainties, I always have the option to call the dispatch center, which I sometimes do, like: hold on, I'm missing a few things."* |
| During transport | Sights into trends | 12 | *“He hasn't deteriorated or anything, in terms of clinical presentation or anything like that, but it is, well, it could be a child that could suddenly change in that PEWS. And that's why I find that PEWS so helpful as well, because you can measure, even unnoticed, for example, that breathing, so I had a saturation monitor on, you know. Then you can follow that a little bit, and then I think: oh, wait a minute, something's going to change there, or am I really seeing correctly what's happening.”* |
|  | Agreements on escalation of care | 10 | *“And it also provides more reassurance that you also know, well, during the transport, if I'll reach certain points, I'll reach that risk, okay, then I know what I need to do. Contact the referring hospital and contact the MMA (Medical Manager Ambulance care).”* |
| **Situational awareness 2** | | | |
| Individual | Objectifying clinical judgment | 6 | *“We naturally have a clinical judgment, that's what I always call it. When you come in, you see a child and you think: hey, is there something wrong or not? But the moment you measure – measuring is knowing – I do think that you become more aware of, oh hey, the child isn't breathing very rapidly, because we would notice that, but it is breathing a bit accelerated. I think it makes you a bit more conscious of your measurements and of how is the child doing now?”* |
|  | Interpretation of vital signs | 4 | *“And that gives you a guideline of what's critical, or what the risks are in this case for a child. Can they be traced back to the event, illness, situation, or what we're doing.”* |
|  | Recognizing deterioration | 4 | *“Having a reference value to look at, like hey, if someone now has a much higher respiratory rate, or a much higher heart rate than what's standard for that age group, I think hey, this is indeed an alarm signal for me. [...] So, it gives me a tool to keep observing the child well, and with any deterioration, because you're conducting those checks, you have a good reference value and you think hey, now I need to sound the alarm, because otherwise, things aren't going well with the child.”* |
|  | Understanding patient’s condition | 4 | *“Because you have very clear references like okay, if at some point I see that the respiratory rate becomes 51, while it should be between 20 and 30, for example, yes, that's an alarm signal for me, and then you start looking further like okay, what comes next. And then you also see that a child is becoming pale, or, well, is in pain, yes, that does give you assistance, yes.”* |

Themes are illustrated by providing quotes from the interviews. All text is translated from Dutch to English. Hesitation has been removed from quotes to enhance readability. SA level 1 and 2 are divided into three sublevels. ‘Individual’ describes particular factors contributing to PEWS, such as the expertise/ experience of healthcare staff and are applied upon arrival as well as during transport. ‘Upon arrival’ describes the effects of PEWS on SA at the moment of handover, prior to transport. ‘During transport’ characterizes the effects of PEWS on SA during transport. N refers to the number of times the response was made in all interviews.

**Table 2.** Results from semi-structured interviews relating to effects on chain of care – before implementation (T=0)

| **Theme** | **Sublevel** | **N** | **Quote** |
| --- | --- | --- | --- |
| **Chain of care** | | | |
| Uniformity | Need for greater uniformity Between hospitals and ambulance services  Within ambulance care | 19  12  7 | *"Well, I think that if there is uniformity, it makes things easier because you communicate on the same level. You talk about the same things, so that would make it a lot easier. Of course, you have it now with PMC (Princes Maxima Center), but it's nice to hear that multiple hospitals are going to use the same PEWS. It prevents irritations, I think, among each other."* |
|  | Speaking the same language  Yes  No | 13  7  6 | *"I definitely think it helps with the communication and handover, because otherwise, we would be looked at like why are you making things complicated because we had different rules than [hospital name]. You would be looked at like, I don't know why you're making it so complicated, but I think you can just transport the child. Yes. It's more consistent now."* |
|  | Not used in the dispatch center | 7 | *"The MEWS score (Modified Early Warning Score) is used there, and when a child is offered, they don't ask for the vital signs at all. And then I think, why not. That is what gives you, because what can happen now? That you come to that institution and then I get the values and I already have 4 points, then I know I can't transport that. So, I have to back out, and another ambulance has to come."* |
| Communication and handovers | Added value of communication Referring institution | 5 | *"It has a significant impact on communication and handover, because, even during handover, everyone, in principle, works with it, and then you recognize each other in the story, and you feel a connection with each other. We speak the same language. And that's just very nice. It feels familiar, and you don't have to explain everything from scratch like, okay, how do you see that then? Everyone speaks the same language."* |
|  | Added value of communication Receiving institution | 2 | *"And it also helps to tell the receiving hospital that the child has remained stable or has shown this and this on the way, so that they can detect it earlier. Because if we didn't do checks, and we are on the road for an hour and a half, we just bring the child to the hospital, say goodbye, and leave. Then they naturally also notice later that the child is deteriorating. Whereas if I've done it all on the way and it still shows a slight deterioration, they can monitor and intervene earlier."* |
| Provider Experience | Attitude towards the PEWS system | 12 | *“Inevitably, it becomes quite tense when you have to transport children at some point. Now, due to the increased emphasis, also in the training, I feel safer and more comfortable, and I believe that I am indeed providing better care now than I did a year ago."* |
|  | Enhancing safety | 8 | *"Yes, I definitely feel more confident in the ambulance when I have to transport a child, even a young child. I've transported a 12-year-old and a 6-year-old at some point, but that was all before this. I think that if I were to transport them now, I would feel more comfortable in the ambulance than I did a year ago when it wasn't really being used. Well, it was there, but it wasn't really emphasized in our practice."* |

Themes are illustrated by providing quotes from the interviews. All text is translated from Dutch to English. Hesitation has been removed from quotes to enhance readability. N refers to the number of times the response was made in all interviews.

**Table 3.** Results from semi-structured interviews relating to effects on the reduction of practice variation – before implementation (T=0)

| **Theme** | **Sublevel** | **N** | **Quote** |
| --- | --- | --- | --- |
| **Reduction of practice variation** | | | |
| Procedures | Clear protocols offer guidance | 19 | *“And besides, it's a good guideline, like, I have these measurements, what can I do with them or what will I do with them, what should I do with them? So, in that respect, it's a good guideline on how to handle a child who is sick or suddenly becomes ill or changes.”* |
|  | Transport decision based on the protocol | 18 | *"Upon arrival, I asked if she had that form for me because this child falls under the pilot. She said no, it's not necessary because this child is too ill for the pilot. I said, 'Okay, but then I might as well leave.' I said, 'But we can also take a look at the child's condition based on the PEWS and see if it can still come with me.' So, we did that, and ultimately, based on the PEWS, it turned out that the child could come with me just fine, so that's a positive experience, that you can turn it around and say, 'The child can come with me just fine.' It was still sick, by the way. It didn't have a score of 0, but..."* |
|  | Escalation of care decisions based on the protocol | 12 | *"And it also provides a bit more peace of mind that you know that, hey, if I reach certain points during the ride, I reach that risk, okay, then I know what to do now. Contact the referring hospital and contact the MMA. Before that pilot program, well, I didn't really know what to do if it happened in the ambulance."* |
|  | Ambiguity in the protocol | 7 | *"And I definitely think that it applies to me already, that it's a significant improvement compared to a year ago […] I find that's still a shortcoming of the employer. We always know for the [organization], we can go up to five points, but it's been unclear whether that also applied to children. I've never received anything in writing about that."* |

Themes are illustrated by providing quotes from the interviews. All text is translated from Dutch to English. Hesitation has been removed from quotes to enhance readability. N refers to the number of times the response was made in all interviews.
