# Supplementary material for: Pediatric Early Warning Score in interhospital ambulance care: a pilot study exploring feasibility and impact
Source: Scand J Trauma Resusc Emerg Med. 2025 Apr 18;33:65. doi: 10.1186/s13049-025-01383-6 (PMC12007274; doi:10.1186/s13049-025-01383-6)
Supplement: Supplementary file 5 — Supplementary Material 5 [file 13049_2025_1383_MOESM5_ESM.docx]

# Additional file 5. Supporting information on results from evaluations

**Table 1.** Results from semi-structured interviews relating to effects on Situational Awareness (SA) – after implementation of the Dutch Ambulance PEWS

| **Theme** | **Sublevel** | **N_total_  (T_6_/T_12_)** | **Quote** |
| --- | --- | --- | --- |
| **Situational awareness 1** | | | |
| Individual | Worried sign  Family  Healthcare provider | 31 (19/12)  20 (11/9)  11 (8/3) | *"And with the parents, well, parents know the child best, of course. And they notice changes very quickly, which is an alarming signal that you need to consider. And that makes it easy for me because it's also described on your Dutch PEWS card. It's a very important item to consider.”* |
|  | Alertness to vital signs | 22 (13/9) | *"That you first take a look at the values, as described on the card, you look at the breathing, the respiratory effort, and whether the child is using oxygen. And if there's a deviation, then you measure the blood pressure. Yes, I find that helpful. I think it's also comforting for the child."* |
|  | Aged-based reference to vital signs | 17 (8/9) | *"It helps me at that moment to look and say, 'I observe a respiratory rate of 34 in the patient,' just as an example, and I can immediately check the age and refer to the Dutch PEWS, and I see that it's normal for the age of that child. Then I know that I don't need to worry about it."* |
|  | Contribution of pocket cards | 12 (7/5) | *"What I do when transporting a child is I look at the age, then I also bring out the card and have it with me so that I can keep an eye on it. And if I see at some point that, hey, it's going up now or it's increasing by one or two points... so, especially that pocket card... yes, it's really a huge tool for us as nurses."* |
| Upon arrival | Risk stratification | 18 (9/10) | *"And also the risk classification... so, your Standard Risk, Medium Risk, High Risk. You have it all at a glance... if you see a red color, well, then everyone knows, guys, this is serious, and green means safe." -* |
|  | Agreements on transport | 4 (3/1) | *"So, we actually want to transport children who are mainly completely stable. And if that's not the case, well, then you have to stay within the PEWS with the respiratory rate and heart rate, and if you go beyond that, well, then you just have to seek help. It's just not safe to transport you anymore."* |
| During transport | Sights into trends | 25 (14/11) | *“Yes, I think it helps to form a trend. In that last trip, that child started with zero... the girl came with us with zero, actually. And halfway through, she scored, well, I think I got to three points, then, of course, she remained in that Standard Risk, so, you have to keep a close eye on it."* |
|  | Risk stratification | 20 (10/10) | *"Well, if I'm transporting a child who falls into the Standard Risk group... then I don't worry so much about it, but if I'm transporting someone who, for example, is in the Medium Risk, then you already know that you have to keep a closer eye on them because they're a bit more threatened, or that the vital functions are more at risk... And that's where you get a nice overview from this."* |
|  | Agreements on escalation of care | 11 (6/5) | *"Well, Dutch PEWS guides you in recognizing deterioration. You just know a next step, from Standard Risk to Medium Risk. Then you just know that there are different actions to be taken. And yes, it's very nice to read that in the action field: you consult with the sending institution's doctor, do your checks every ten minutes, your checks become more intensive. I think Dutch PEWS provides clarity in that regard […] And it also gives you peace of mind, especially if the deterioration continues, and you know that in the High Risk, you should call for an A-level (High Complex) ambulance to come."* |
| **Situational awareness 2** | | | |
| Individual | Objectifying clinical judgment | 13 (5/8) | *"It provides numbers for what you see and know. And it's more of an additional confirmation and a tool to see, 'Hey, I have this now, does it match what I see, experience, and know?'"* |
|  | Interpretation of vital signs | 10 (4/6) | *"Well, at a certain point, you can also see, 'Hey, my heart rate is now going towards 140... you can see that immediately on your monitor. So, it's nice to have that card alongside and think, 'Hey, something must be going on now. Because why would a heart rate go to 140 or something like that?'"* |
|  | Understanding patient’s condition | 6 (2/4) | *"And by looking at, 'Okay, I see rapid breathing, is it indeed so fast that I should be worried because how many points does it score, 1 or 2 points? Or maybe more? Well, there are other values like that. Yes, I get a good picture from that, and whether I need to intervene."* |
|  | Worried sign – family | 5 (1/4) | *"Well, I think with Dutch PEWS, for us - especially low-care oriented - it... well, the family is more involved... or, at least, the father/mother/guardian is more involved. SP1: And how does that help you? SP2: Well, look, we might have a stable patient, a stable child in this case, but if the parent is still concerned or anything, well, with this new Dutch PEWS, it raises the level a bit, and it makes us think, 'Did we really examine it well? Is the decision being made correctly, and is it indeed safe? Is it responsible to proceed with this trip?'”* |
|  | Recognizing deterioration | 4 (2/2) | *" I think it would recognize such deterioration in time. At least by measuring the values. It's very clear, well, good, then you switch to a different score. Yes, it contributes. The concern of the mother or father obviously plays a role in it. Yes, it gives me the idea of recognizing a problem that's beginning to arise. Also, if a child, for example, becomes less alert, well, those are signs that go into it. And in that regard, I find Dutch PEWS very clear. Well, when a child is alert and then, at some point, scores one of the others... 'verbal' or 'pain' or no response anymore, then you're immediately in that High Risk. And that's clear. A child that, at some point, starts to have reduced consciousness, well, that, by definition, just means calling an A-level ambulance."* |
| Upon arrival | Consultation with MMA (Medical Manager Ambulance care) regarding interpretation of condition | 8 (5/3) | *"But, well, that's up to the doctor. And it's done in consultation. And if I were to have doubts, say if a doctor says a child can be transported because these values belong to the child, then I think I would still consult with my medical manager. Like, 'Hey, I'm here now, I'm in this Medium Risk, but we can proceed with responsible transport, I also feel comfortable. And that's, of course, my peace of mind or reassurance.'"* |
| During transport | Interpretation of trend break | 11 (3/8) | *"I can handover a child with the values I have, and say, 'The child falls into this or that category.' If I have a child that's on the edge, suppose just before arrival, the mother becomes concerned, then I would say, 'Well, during the trip, it was standard risk, but the mother is getting a bit concerned now, so actually, we're moving towards medium risk.'"* |

Themes are illustrated by providing quotes from the interviews. All text is translated from Dutch to English. Hesitation has been removed from quotes to enhance readability. SA level 1 and 2 are divided into three sublevels. ‘Individual’ describes particular factors contributing to PEWS, such as the expertise/ experience of healthcare staff and are applied upon arrival as well as during transport. ‘Upon arrival’ describes the effects of PEWS on SA at the moment of handover, prior to transport. ‘During transport’ characterizes the effects of PEWS on SA during transport. N_total_ refers to the total number of times the response was made in all interviews and is subsequently divided into the number of times the response was made in T=6 and T=12.

SP1 refers to speaker one (interviewer), SP2 refers to speaker two (participant).

**Table 2.** Results from semi-structured interviews relating to effects on chain of care – after implementation

| **Theme** | **Sublevel** | **N_total_  (T_6_/T_12_)** | **Quote** |
| --- | --- | --- | --- |
| **Chain of care** | | | |
| Uniformity | Speaking the same language  Yes  No | 28 (14/14)  21 (9/12)  7 (5/2) | *"But a few months ago, I had to transport a child from WKZ (Wilhelmina Children’s hospital) to [hospital] in [place] and I found it very nice to see that both in WKZ and in [hospital], they were both aware of the Dutch PEWS. […] Then you know what it's about, so both the nurse in [place] and in WKZ, and also me. And then you are all on the same page with each other."* |
|  | Need for greater uniformity Between hospitals and ambulance services  Within ambulance care | 22 (8/14)  13 (4/9)  9 (4/5) | *"I think as more hospitals start working with it, it does provide added value for the ambulance service in the Netherlands because it provides a clear policy, instead of using various different scoring systems and getting different handovers with 'oh wait a minute, what do you mean exactly?', and that just creates confusion and sometimes uncertainty, and if you just have one system, both in the ambulance service and in pediatric hospitals, it provides much more clarity."* |
|  | Utilization in hospitals  Yes  No | 21 (7/14)  12 (3/9)  9 (4/5) | *"And, well, what I like is that, especially when you're from [place], [organization] or [organization], everyone works with the Dutch PEWS, and you know what you're talking about with each other."*  *"I noticed in the past year that certain hospitals didn't use it and they used a different PEWS, yes, then you're not on the same page and also that some had no knowledge at all about the Dutch PEWS. Yes, that caused confusion for us too."* |
|  | Clarity regarding agreements within the chain of care | 6 (0/6) | *"SP1: Why is it nice that the hospital also uses the Dutch PEWS?*  *SP2: You can explain it better. The communication between each other is clearer. Why do you have a Medium Risk? What is the reason for that, what do you need to do then? Why are you suddenly calling a doctor?"* |
| Communication and handovers | Added value of communication Receiving institution | 16 (4/12) | *" If I have a child that's on the edge, suppose just before arrival, the mother becomes worried, then I will say well during the trip it was standard risk, but now mother is starting to worry a bit so actually we are moving towards medium risk now. [...] Yes, I can transfer very well and especially if they speak the same language."* |
|  | Contribution to uniform communication | 12 (8/4) | *"SP1: Yes, because you say, we speak the same language... what is the effect of speaking the same language and being on the same page for you?*  *SP2: Yes, that the chance of errors and misinterpretations is much smaller. And the chance of misinterpretations and errors that come with it. Or that one might interpret it differently than I mean it. That's how I see it, actually."* |
|  | Added value of communication Referring institution | 6 (2/4) | *"Well, I think we are now aligned with hospitals. [….] The nurse I had; she knew about the Dutch PEWS. And it matched very nicely. And speaking the same language, having the same values, and talking about the same risk group. You think more in those three risk groups now. Because it actually summarizes it. I experience that as a big improvement."* |
|  | Added value of risk stratification in communication | 7 (5/2) | *“That you essentially speak the same language everywhere, so you know exactly, for example, with a Standard Risk, I don't have to do much, but if it's a Medium Risk or a High Risk, then we know everywhere what you should do. And that's the advantage of it. So that you recognize the threatened vital functions. And that immediate actions can be taken. That they are already described."* |

Themes are illustrated by providing quotes from the interviews. All text is translated from Dutch to English. Hesitation has been removed from quotes to enhance readability. N_total_ refers to the total number of times the response was made in all interviews and is subsequently divided into the number of times the response was made in T=6 and T=12.

SP1 refers to speaker one (interviewer), SP2 refers to speaker two (participant).

**Table 3.** Results from semi-structured interviews relating to effects on the reduction of practice variation – after implementation

| **Theme** | **Sublevel** | **N_total_  (T_3_/T_6_/T_12_)** | **Quote** |
| --- | --- | --- | --- |
| **Reduction practice variation** | | | |
| Protocol adherence | Clear protocols offer guidance | 62 (18/22/22) | *" It just makes you more confident when you transport. You know exactly what you can do and what actions you need to take. I find that pleasant. I have just gained more certainty through this."* |
|  | Escalation of care decisions based on the protocol | 28 (6/12/10) | *"And if you see signs that a child is deteriorating, that you then also do your checks at that moment and that you eventually come to something. And then it exactly states what you should do or with whom you should consult or can consult. That makes it pleasant."* |
|  | Adhering to protocol | 24 (8/5/11) | *"By adhering to the actions for the different risk groups, so if you are in the Standard Risk, that is your starting position, just do those checks every half hour. And if you see a change, yes, then you move on to the corresponding actions of that risk... if you go to Medium Risk, then you measure every ten minutes. Or you may have a feeling along the way, hey, this isn't going well, so I need to do an extra check."* |
|  | Transport decision based on the protocol. | 21 (8/ 4/9) | *"And with the Dutch PEWS you have very clearly, your Standard Risk, your Medium Risk, and your High Risk. And the points are corresponded with that. And especially for us as ambulance personnel, it just makes it very easy to say, well, it scores this number of points, so I'll transport this child or I won't transport the child."* |
|  | Utilization of pocket cards | 19 (11/4/4) | *“That I have certainty, that I know that I can escalate if necessary. And I want to know what steps I need to take. And if I don't remember, then I can look at my card. And that is nice. I can imagine that the situation might be panicky, with the parents present, then you have to take the right steps [...] So in that respect, I find it a nice common thread that runs through it, which you can always consult."* |
| Dissemination | Applicability within the operational procedures of Medium Complex ambulances. | 21 (11/0/10) | *"Well, I think with the introduction now done and with the materials we have, and there are of course other ambulance services in the Netherlands, that they can also provide safe care. So that it is also very applicable to other ambulance services."* |
|  | Applicability within the operational procedures of High Complex ambulances.  Yes  No | 5 (5/0/0)  3 (2/0/1) | *"SP1: And do you think that it could be used for other forms of inter-hospital transport or for high complexity or low complexity?*  *SP2: Yes, I think so. As long as we all continue to speak the same language. If I have to take over something from someone else or if I have to be taken over by someone else, that we understand why that would be."*  *"I wonder if it has added value for the rapid response car. Because they work in their own way, they just work differently. They don't work with that EWS (Early Warning Score), so not with PEWS either. I wonder about that. I think it is more suitable for the B-car (medium and low complex ambulances). I would advocate to train the low complexity cars."* |
|  | Applicability within the operational procedures of Low Complex ambulances.  Yes  No | 10 (7/0/3)  3 (0/0/3) | *"Yes, definitely usable for other ambulance services. I don't think for high complexity because they work in a slightly different way, but I think for low and medium complexity, it is certainly usable."*  *"I do notice that with low complexity, and there you mainly deal with caregivers, so they are not nurses, they find it tense and they also find it difficult to use such a card. They would rather not have to transport a young child because they find that tense. Whereas with medium complexity, that's not the case. So, I think with medium complexity, it's well-received, but with low complexity, a bit less."* |

Themes are illustrated by providing quotes from the interviews. All text is translated from Dutch to English. Hesitation has been removed from quotes to enhance readability. N_total_ refers to the total number of times the response was made in all interviews and is subsequently divided into the number of times the response was made in T=3, T=6 and T=12.

SP1 refers to speaker one (interviewer), SP2 refers to speaker two (participant).

**Table 4.** Results from semi-structured interviews relating to implementation of the DA-PEWS

| **Theme** | **Sublevel** | **N_total_  (T_3_/T_6_/T_12_)** |
| --- | --- | --- |
| **Implementation** | | |
| Barriers | Frequency of pediatric transfers | 24 (7/6/11) |
|  | Background knowledge on protocol | 11 (0/5/6) |
|  | Various working methods | 7 (3/1/3) |
|  | Anxiety related to pediatric transfers | 5 (3/0/2) |
|  | Transition and confusion with new system | 5 (5/0/0) |
|  | Education training | 4 (2/2/0) |
| Facilitators | Educational training | 25 (14/3/8) |
|  | Pocket cards | 20 (9/5/6) |
|  | User-friendliness | 12 (4/5/3) |
|  | Understanding of protocol | 11 (4/7/0) |
|  | Researchers’ involvement in educational training | 6 (6/0/0) |
|  | Implementation materials | 6 (6/0/0) |
|  | Motivating staff | 4 (4/0/0) |
| Provider Experience | Positive attitude towards transition | 30 (19/5/6) |
|  | Positive attitude towards D-PEWS | 29 (9/4/16) |
|  | Clarity in protocol  Yes  No | 28 (12/8/8) 3 (3/0/0) |
| Recommendations for implementation | Using more practical sessions during educational training | 5 (5/0/1) |

N_total_ refers to the total number of times the response was made in all interviews and is subsequently divided into the number of times the response was made in T=3, T=6 and T=12. The barrier ‘various working methods’ includes regional differences between ambulance services as well as hospitals using a different PEWS-system, resulting in confusing whether or not to apply the Dutch PEWS.
